# Ghrelin deletion protects against age‐associated hepatic steatosis by downregulating the C/EBPα‐p300/DGAT1 pathway

**DOI:** 10.1111/acel.12688

**Published:** 2017-10-12

**Authors:** Bobby Guillory, Nicole Jawanmardi, Polina Iakova, Barbara Anderson, Pu Zang, Nikolai A. Timchenko, Jose M. Garcia

**Affiliations:** ^1^ Department of Medicine Baylor College of Medicine Division of Endocrinology Diabetes and Metabolism, MCL Center for Translational Research in Inflammatory Diseases Michael E. DeBakey Veterans Affairs Medical Center Houston TX 77030 USA; ^2^ Huffington Center on Aging Baylor College of Medicine Houston TX 77030 USA; ^3^ Department of Pathology and Immunology Baylor College of Medicine Houston TX 77030 USA; ^4^ GRECC VA Puget Sound Health Care System University of Washington Seattle WA 98108 USA; ^5^ Department of Endocrinology Nanjing Jinling Hospital Nanjing 210002 China; ^6^ Cincinnati Children's Hospital Medical Center Cincinnati OH 45229 USA

**Keywords:** aging, C/EBP proteins, ghrelin, liver, p300, steatosis

## Abstract

Nonalcoholic fatty liver disease (NAFLD) is the most common liver disease worldwide. NAFLD usually begins as low‐grade hepatic steatosis which further progresses in an age‐dependent manner to nonalcoholic steatohepatitis (NASH), fibrosis, cirrhosis, and hepatocellular carcinoma in some patients. Ghrelin is a hormone known to promote adiposity in rodents and humans, but its potential role in hepatic steatosis is unknown. We hypothesized that genetic ghrelin deletion will protect against the development of age‐related hepatic steatosis. To examine this hypothesis, we utilized ghrelin knockout (KO) mice. Although no different in young animals (3 months old), we found that at 20 months of age, ghrelin KO mice have significantly reduced hepatic steatosis compared to aged‐matched wild‐type (WT) mice. Examination of molecular pathways by which deletion of ghrelin reduces steatosis showed that the increase in expression of diacylglycerol O‐acyltransferase‐1 (DGAT1), one of the key enzymes of triglyceride (TG) synthesis, seen with age in WT mice, is not present in KO mice. This was due to the lack of activation of CCAAT/enhancer binding protein‐alpha (C/EBPα) protein and subsequent reduction of C/EBPα‐p300 complexes. These complexes were abundant in livers of old WT mice and were bound to and activated the DGAT1 promoter. However, the C/EBPα‐p300 complexes were not detected on the DGAT1 promoter in livers of old KO mice resulting in lower levels of the enzyme. In conclusion, these studies demonstrate the mechanism by which ghrelin deletion prevents age‐associated hepatic steatosis and suggest that targeting this pathway may offer therapeutic benefit for NAFLD.

## Introduction

Nonalcoholic fatty liver disease (NAFLD) is very prevalent, particularly in the aging population. It is the most common cause of cirrhosis worldwide (Lazo & Clark, 2008) and is strongly associated with the development of diabetes, obesity, hypertension, and dyslipidemia (Browning *et al*., 2004a,b). The impact of NAFLD as a cause of liver cancer is projected to expand in coming years as the rate of obesity (Riordan & Nadeau, 2014) and the aging segment of the population increase (2008).

Nonalcoholic fatty liver disease is characterized by increased triglyceride accumulation in the cytoplasm of hepatocytes, but despite intensive investigations, the mechanisms leading to the development of fatty liver have not been completely characterized (Brunt, 2010). NAFLD is generally asymptomatic and usually diagnosed as advanced disease upon initial examination. To date, there are no clinical recognized biomarkers validated for the diagnosis of NAFLD. Furthermore, there are no available treatments for this condition. Hepatic steatosis is the primary step leading to NAFLD that may progress to steatohepatitis and cirrhosis. Our study suggested a possible mechanism for age‐associated steatosis which includes activation of the transcription factors p300‐C/EBPα/β complexes that turns on promoters of five genes that are responsible for driving triglyceride synthesis (Jin *et al*., 2013). Moreover, we recently demonstrated that the inhibition of this pathway prevents development of hepatic steatosis and that its inhibition in mice with existing steatosis reverses this process (Jin *et al*., 2016).

Ghrelin, an orexigenic peptide and the endogenous ligand for the growth hormone secretagogue receptor 1a (GHSR‐1a), is a novel hormone that increases GH secretion and adiposity in humans and rodents (Tschop *et al*., 2000). However, its role on hepatic steatosis is not known. Recent publications have suggested that it may increase liver adiposity through p53 signaling (Davies *et al*., 2009; Porteiro *et al*., 2013), although others have shown the opposite effect with ghrelin preventing fat accumulation and improving redox state in the liver of high fat‐fed animals (Barazzoni *et al*., 2013) and also under pair‐feed conditions (Stark *et al*., 2015). The goal of this work was to determine whether deletion of ghrelin might prevent the age‐associated NAFLD and to determine whether its effects are mediated through the p300‐C/EBPα/β pathway.

## Results

### Ghrelin deletion prevents age‐related increase in liver weight and hepatic steatosis

**Figure 1:**
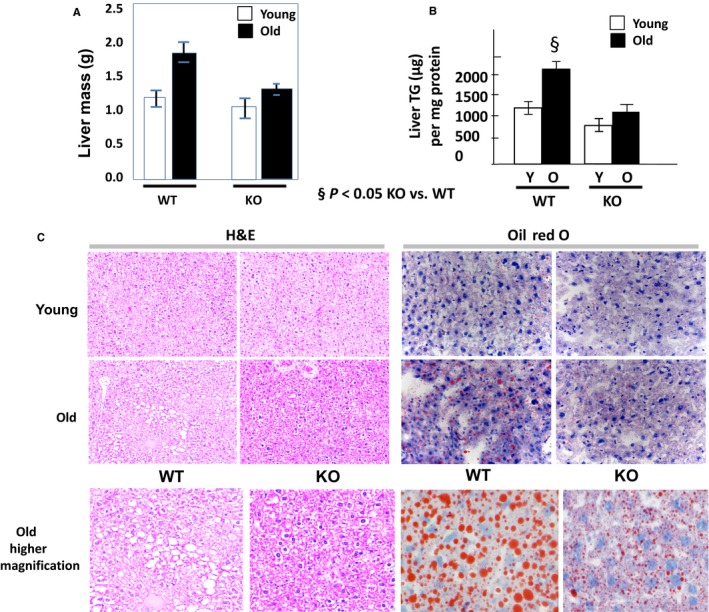
Liver lipid accumulation in young and old ghrelin wild‐type and knockout animals. (A) Liver mass and (B) liver triglyceride (TG) content by mg of protein. (C) 20× H&E sections of young and old, WT, and KO liver tissue; 40× oil red O staining counter stained with hematoxylin. Detail of 40× oil red O staining counter stained with hematoxylin in sections of old WT and KO liver tissue (below). WT: wild‐type, KO: knockout. ^§^ *P* < 0.05 KO vs. WT.

Liver mass was measured in young (100 days old) and old (600 days old) ghrelin wild‐type (WT) and knockout (KO) c57bl/6j male mice (*n *=* *8/group) upon dissection, and liver fat content was ascertained by directly measuring triglyceride (TG) content in liver tissue. Liver mass tended to increase with age and to be smaller in KO compared with WT animals, even though these differences did not reach statistical significance (Fig. 1A). Liver TG tended to increase with age in both genotypes but was significantly lower in old KO mice than in WT (Fig. 1B). Hepatic hematoxylin and eosin (H&E) and oil red O (ORO) staining showed almost no fat droplets in both genotypes in young animals. However, there was a significant increase in fat droplets with aging seen in WT animals that was not detected in the KO (Fig. 1C). Body weight was similar between young WT and KO mice (27.4 ± 0.09 g and 28.6 ± 0.82 g, respectively, *P* value NS); however, it increased significantly with age in WT but not in KO animals (43 ± 4.46 g and 32.7 ± 1.22 g, respectively, *P* < 0.05). Young ghrelin WT mice had higher food intake compared to KO (daily food intake/body weight: 14.5 ± 0.4 g/100 g in WT, 10.8 ± 0.2 g/100 g in KO, *P* < 0.005), although this difference was not seen in aged mice (daily food intake/body weight: 8.7 ± 0.7 g/100 g in WT, 8.6 ± 0.7 g/100 g in KO, *P* > 0.9). Taken together, the data suggest that ghrelin deletion prevents the development of age‐associated hepatic steatosis.

### Ghrelin deletion prevents age‐dependent upregulation of hepatic DGAT1

**Figure 2:**
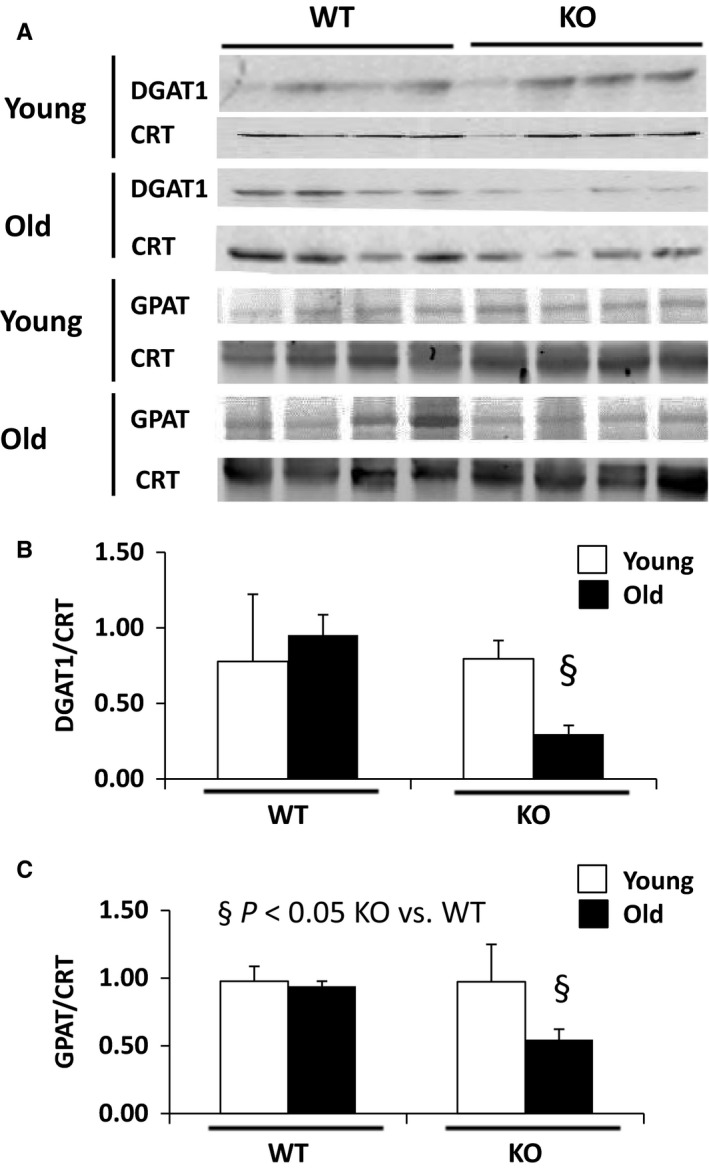
Ghrelin deletion prevents the increases in glycerol‐3‐phosphate acyltransferase (GPAT) and acyl CoA:diacylglycerol acyltransferase‐1 (DGAT1) expression seen with aging. (A) Western blot analysis probed with anti‐GPAT and DGAT, (B) Densitometric calculations of DGAT1 and (C) GPAT. CRT: calreticulin, ^§^ *P* < 0.05 KO vs. WT.

As triglycerides are the main components of fat droplets, we investigated the pathways regulating hepatic triglyceride synthesis. We measured the expression of glycerol‐3‐phosphate acyltransferase (GPAT) and acyl CoA:diacylglycerol acyltransferase‐1 (DGAT1), two key enzymes which regulate the first and last steps of TG synthesis, respectively. Western blotting analyses revealed that protein levels for DGAT1 are increased with age in livers of WT mice in agreement with previous observations (Jin *et al*., 2013), whereas GPAT remained stable. However, old ghrelin KO mice had lower GPAT and DGAT1 protein levels than their WT counterparts (Fig. 2A–C). The potential contribution of other pathways known to regulate fatty acid biosynthesis and oxidation in liver was explored by measuring by RT–PCR and Western blotting, the transcript and protein levels of key mediators including peroxisome proliferator‐activated receptor alpha (PPAR‐α), fatty acid synthase (FAS, a downstream mediator of sterol regulatory element binding protein [SREBP] 1c), lipoprotein lipase (LPL), Stearoyl‐CoA desaturase‐1 (SCD‐1), and liver adipose TG lipase (ATGL). No significant differences were found between groups for any of these transcripts/proteins (Fig. S1‐S2).

### Deletion of ghrelin changes expression and phosphorylation of CCAAT‐enhancer‐binding protein (C/EBP) α

**Figure 3:**
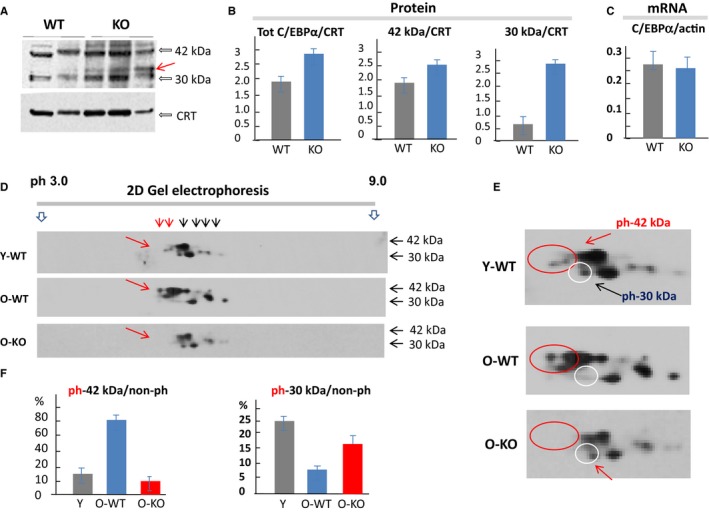
Transcription factor C/EBPα analysis using Western blot shows increased expression in older ghrelin knockout animals. (A) Isolated proteins from liver tissue of old animals were separated by gel electrophoresis and probed with anti‐C/EBPα. Red arrow indicates the 30‐kD isoform with different electrophoretical mobility. (B) Densitometric calculations of C/EBPα of the 30 and 42 mouse isoforms. (C) C/EBPα mRNA levels measured by RT–PCR. (D) Two‐dimensional technique gel analysis of C/EBPα isoforms expression in young wild‐type, old wild‐type, and old knockout animals. (E) Enlargement of representative sections from (D), the red circle represents the gel region where the phosphorylated protein ph‐42kD is observed and the white circle is the region of the gel where S193‐ph‐30kD is observed. (F) Densitometric calculations of 2D gel analysis. WT: wild‐type, KO: knockout, CRT: calreticulin, Y: young, O: old.

C/EBPα is a transcription factor belonging to the C/EBP family that regulates a number of biological processes in the liver including TG synthesis, insulin response, and energy metabolism (Park *et al*., 1990, 1993; Yanuka‐Kashles *et al*., 1994; Timchenko, 2009). It has been shown that the ph‐S193 isoform of C/EBPα preferentially interacts with p300 and activates promoters of enzymes of TG synthesis including DGAT1 (Jin *et al*., 2013, 2016). Aging activates this pathway by inducing C/EBPα phosphorylation at Ser193 (Timchenko *et al*., 2006) and the formation of C/EBPα‐p300 complexes (Jin *et al*., 2013, 2016). Therefore, we proposed that this pathway might be inhibited during aging by the deletion of ghrelin. To test this hypothesis, we initially examined expression of C/EBPα using RT–PCR, Western blotting, and 2D gel electrophoresis techniques. C/EBPα is expressed in the mouse liver as two isoforms with molecular weights 42 and 30 kD. Although there was no difference in transcript levels of C/EBPα by RT–PCR, the comparison of these isoforms in livers from ghrelin KO and WT mice by Western blotting showed that the total levels of both isoforms are slightly higher in the KO mice. Importantly, the truncated 30‐kD isoform also changed its electrophoretical mobility suggesting an increase in phosphorylation. Densitometric calculations revealed that both isoforms of C/EBPα are increased in livers of KO mice; however, 30‐kD isoform is increased much more, leading to a change in the ratio of 30‐kD to 42‐kD isoforms (Fig. 3A–C). The ability of C/EBPα to form complexes with p300 mainly depends on phosphorylation of C/EBPα at Ser193. Using knock‐in animal models, we have shown that C/EBPα de‐phosphorylated at Ser193 does not form C/EBPα‐p3000 complexes (Jin *et al*., 2015). To examine phosphorylation of C/EBPα in old ghrelin KO mice, we next applied the 2D technique and compared C/EBPα isoforms in WT young, WT old and KO old mice. A typical picture of 2D‐Western blotting is shown in Fig. 3D, and the enlarged images are presented in Fig. 3E. As one can see, livers of WT old mice contain high amounts of 42kD‐S193ph isoform, but 30kD‐Ser‐139‐ph isoform is not detectable in old WT animals. Quite different pattern of C/EBPα isoforms is observed in livers of old KO mice resembling that of young WT animals with low expression levels of 42kD‐Ser193‐ph isoform of C/EBPα whereas levels of the 30kD‐ph isoform are restored. Densitometric calculations revealed that livers in KO mice fail to accumulate 42kD S‐193‐ph isoform of C/EBPα, but have increased truncated 30kD C/EBPα phosphorylated at Ser193 (Fig. 3F). Thus, these studies showed a significant difference in C/EBPα between old WT and KO mice and suggest that modulation of this pathway by ghrelin plays a role in the lack of liver fat accumulation in this setting.

### Livers of KO mice do not accumulate C/EBPα‐p300 complexes with age leading to the lack of activation of DGAT1 promoter

**Figure 4:**
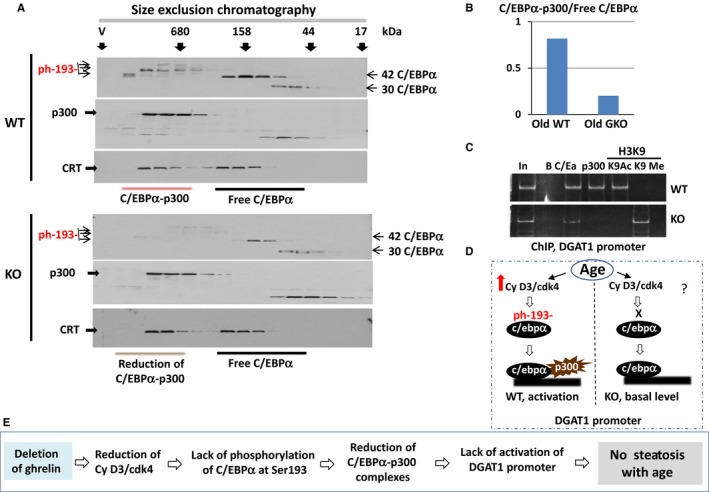
C/EBPa‐p300 complexes are reduced in livers of GKO mice leading to a lack of activation of DGAT1 promoter. (A) Nuclear extracts from old WT and GKO mice were separated by size exclusion chromatography (SEC), and location of C/EBPα and p300 proteins was determined using Western blotting assay. The membranes were reprobed with antibodies to calreticulin. Positions of markers of SEC are shown on the top. Positions of 42kD and 30‐kD isoforms of C/EBPα are shown by arrows. (B) Densitometric calculations of ratios of C/EBPα in complexes wit p300 and free C/EBPα. (C) ChIP assay with DGAT1 promoter. C/EBPα, p300, histone H3K9‐Ac, and histone H3K9‐tri met were immunoprecipitated from chromatin solutions of old WT and GKO mice, and IPs were analyzed by RT–PCR with primers covering C/EBP site within the DGAT1 promoter. (D) A diagram summarizing data of ChIP assay. (E) A hypothesis for the lack of age‐associated hepatic steatosis in GKO mice (see text).

As phosphorylation of C/EBPα at Ser193 is critical for formation of C/EBPα‐p300 complexes and because C/EBPα‐p300 complexes activate promoters of enzymes of TG synthesis, we next examined these complexes in livers of old WT and KO mice. For this goal, we have applied HPLC‐based size exclusion chromatography (SEC). Nuclear extracts were separated by the SEC400 column, and the fractions were tested by Western blotting with antibodies to C/EBPα and to p300. We also performed Western blotting with antibodies to calreticulin (CRT) to ensure that separation of total proteins is identical in these two runs. The results are shown in Fig. 4A. In livers of old WT mice, a significant portion of 42kD C/EBPα is detected in high molecular weight (HMW) fractions, while a very minor portion of C/EBPα is observed in these fractions of the chromatography with proteins from livers of old KO mice. It is important to note that 42 kD C/EBPα is observed in HMW fractions of old WT mice as multiple isoforms which perhaps reflect the hyperphosphorylation status of C/EBPα. Examination of p300 revealed that p300 colocalizes with 42kD C/EBPα in all HMW fractions suggesting that these fractions contain C/EBPα‐p300 complexes. Calculations of the amounts of C/EBPα colocalized with p300 in HMW fractions showed that up to 80% of 42kD C/EBPα is observed in fractions with p300 in WT animals, while less than 20% of C/EBPα from KO livers is detected in the fractions with p300 (Fig. 4B). Thus, these studies show that the amounts of C/EBPα‐p300 complexes are dramatically reduced in livers of old KO mice compared to those in livers of old WT mice.

We have previously shown that the DGAT1 promoter contains a C/EBP binding site and may be activated by C/EBPα‐p300 complexes (Jin *et al*., 2013); and so we next examined occupation of DGAT1 promoter by C/EBPα‐p300 complexes in livers of old WT mice and old KO mice using chromatin immunoprecipitation (ChIP) approach. Moreover, we also examined modifications of histone H3 at lysine K9 to determine the status of the DGAT1 promoter. It has been shown that acetylation of H3 at K9 activates the promoters, while trimethylation at K9 represses the promoters (Stewart *et al*., 2005). The results of these studies are shown in Fig. 4C. We found that DGAT1 promoter is occupied by the C/EBPα‐p300 complexes in livers of old WT mice; however, p300 is not detected on the promoter in livers of old KO mice. Consistent with this pattern of binding, the DGAT1 promoter is activated in old WT mice because the histone H3 is acetylated at K9. On the contrary, the major portion of H3 is trimethylated at K9 showing that the promoter is not activated and it is partially repressed in old KO mice. It is worth noting that we can detect H3K9 acetylation on the DGAT1 promoter in KO mice if we use bigger amounts of chromatin solution showing that the promoter is partially repressed and partially activated. Based on these studies of C/EBPα‐p300 complexes and data of ChIP assay, we conclude that the C/EBPα‐p300 complexes are not accumulated in old KO mice and that this leads to the lack of activation of the DGAT1 promoter and prevention of hepatic steatosis associated with aging. It has been shown that cyclin D3‐cdk4 is activated in livers of old WT mice and phosphorylates C/EBPα at Ser193 (Wang *et al*., 2006). Therefore, we suggest that the reduction of phosphorylation of C/EBPα in livers of GKO mice might be associated with lack of activation of cdk4 (Fig. 4D–E).

## Discussion

Nonalcoholic fatty liver disease (NAFLD) is a very prevalent and relevant condition. It can lead to cirrhosis and liver cancer and is overrepresented in individuals over the age of 65. The first step in the development of NAFLD is hepatic steatosis, and this condition is also very common in the elderly. In spite of its significance, the molecular mechanisms underlying hepatic steatosis are incompletely understood and treatment options for this disease remain elusive. Recent data from our group showed that TG synthesis is an important pathway in the development of age‐related hepatic steatosis and in patients with NAFLD and that a critical regulator of this pathway is the p300‐C/EBPα/β complex that binds and activates the promoters for several enzymes in the TG synthesis pathway, including DGAT1 (Jin *et al*., 2013, 2016).

Ghrelin is the endogenous ligand for the growth hormone secretagogue receptor 1a (GHSR‐1a) and is known to cause fat accumulation by increasing food intake and by other food intake‐independent pathways (Murphy *et al*., 1998; Tschop *et al*., 2000; Nakazato *et al*., 2001), in spite of aging not being associated with significant changes in circulating ghrelin levels or tissue ghrelin receptor expression (Sun *et al*., 2007). Ghrelin is primarily produced in the stomach (~70%) although other tissues including the liver also produce ghrelin (Sun *et al*., 2003). However, the only ghrelin receptor known to this date (GHSr‐1a) is not present in liver tissue (Sun *et al*., 2007). An indirect effect of ghrelin in the liver mediated through GH secretion has been proposed before (Zhang *et al*., 2015). Also, the vagus nerve has been shown to mediate some of ghrelin's effects via the ghrelin receptor GHSR‐1a (Iwasaki *et al*., 2015). Recently, an alternative—yet unidentified—receptor has been proposed as a mechanism for ghrelin to exert effects *in vitro* in hepatocytes, myocytes, or adipocytes (Chen *et al*., 2015). The exact mechanism mediating ghrelin's effects in liver is not well characterized and should be the subject of future studies.

Several reports explored the effects of ghrelin on fat metabolism in the liver, but its role remains controversial. Expression of the key element in the fatty acid synthesis pathway SCD1 mRNA increased, but acetyl‐CoA carboxylase (ACC), fatty acid synthase (FAS), and carnitine palmitoyl transferase (CPT)‐1α mRNAs were unchanged in one study where ghrelin was administered (Theander‐Carrillo *et al*., 2006). In another report, a low dose of ghrelin administered peripherally produced an increase in FAS and decreases in CPT‐1α mRNAs expression without affecting food intake. Although some reports have shown an increase in liver adiposity with ghrelin treatment (Davies *et al*., 2009), others have shown the opposite effect with ghrelin preventing fat accumulation and improving redox state in the liver of high fat‐fed animals (Barazzoni *et al*., 2013) but also under caloric restriction (Stark *et al*., 2015). Additional factors may account for the differences in results reported by different groups: mouse age and strain, subchronic vs. chronic ghrelin treatment and via of administration (central vs. peripheral).

We took a different approach and sought to characterize the role of ghrelin by studying young and old mice with a genetic deletion of the ghrelin gene and their wild‐type counterparts. These mice were established in a C57bl background, which is susceptible to develop hepatic steatosis, obesity, and insulin resistance with age (Neuschwander‐Tetri, 2005; Anstee & Goldin, 2006; Schattenberg & Galle, 2010). Indeed, wild‐type animals developed hepatic steatosis as they aged and KO animals were protected from this as we hypothesized. As we have shown, upregulation of key enzymes on the TG synthesis pathway plays a central role in the development of hepatic steatosis associated with aging (Jin *et al*., 2013, 2016); therefore, we tested whether this pathway was involved in the prevention of this condition in ghrelin KO animals. Levels of the key enzymes DGAT1 were significantly upregulated with aging in WT animals but not in the KO, suggesting that ghrelin deletion prevents aging‐associated hepatic steatosis by downregulating this pathway. Furthermore, the p300‐C/EBPα/β complex that binds and activates the promoter for DGAT1 was also downregulated in old KO when compared to WT animals, confirming that this pathway is affected in this setting. Our studies also detected elevation of C/EBPα protein in GKO mice; while C/EBPα mRNA was not changed. We have previously showed that Ser193 phosphorylated C/EBPα is degraded by the ubiquitin–proteasome system (UPS); however, de‐phSe193 C/EBPα is resistant to this degradation (Wang *et al*., 2010; Jiang *et al*., 2013). We propose that the increase in total C/EBPα protein we saw in old KO mice is due to de‐ph‐C/EBPα not being degraded in GKO.

Multiple pathways are known to regulate fat metabolism in the liver. This work focused on the C/EBPα pathway because our previous experiments showed that hepatic C/EBPα phosphorylation is increased by ghrelin (Wang *et al*., 2007). Although the contribution of other pathways was not characterized here in detail, the fact that transcript and protein levels for mediators in these pathways were similar between groups suggests that they do not play a major role in mediating the effects of ghrelin deletion in this setting. More studies will be needed to explore this issue further. Although a potential contribution of lower food intake to the effects of ghrelin deletion in this setting cannot be ruled out, it is unlikely to be a major contributor because old animals in both genotypes had similar food intake, and because lower food intake does not influence accumulation of fatty acids in the liver (Bruss *et al*., 2010).

Taken together, current evidence suggests that the physiologic role of ghrelin may involve a switch toward anabolism and energy storage preparing the body to deal with negative caloric balance (Zhang *et al*., 2015). This study demonstrates that ghrelin deletion prevents age‐associated hepatic steatosis through downregulation of the p300/C‐EBPα/β/DGAT1 pathway. Furthermore, the data suggests that targeting this pathway may offer therapeutic benefit for NAFLD.

## Experimental procedures

### Animal experiments

All experiments were conducted on adult male C57bl/6J WT, *ghrelin* ^*+/+*^, and KO, *ghrelin* ^−*/*−^ mice obtained from Dr. Roy G. Smith Ph.D's laboratory (Sun *et al*., 2003). As previously published, mouse ghrelin genomic DNA clones were isolated with exon 4‐ and exon 5‐specific primers. The LacZ/Neo selection cassette was inserted into the ghrelin locus to replace ghrelin exons 2 and 3 that encode ghrelin. The targeted ES cells and mice were genotyped by Southern analysis. The ghrelin exon‐specific probe detected a signal in wild‐type and heterozygotes but not in homozygotes. Genotype was confirmed by RT–PCR, and these *ghrelin* ^−*/*−^ mice have been shown to have undetectable serum ghrelin levels and ghrelin mRNA transcript in brain and stomach tissue. Animals were individually housed for 1 week prior to and during the experiments and exposed to 12/12 light/dark cycle (lights on at 6AM). Mice were fed standard nonsoy rodent chow (Advanced Protocol PicoLab Cat#: 3002906‐203, 5V5R) throughout the study. All experiments were conducted with the approval of the Institutional Animal Care and Use Committee and were in compliance with the NIH Guidelines for Use and Care of Laboratory Animals. Experimental animal numbers ranged from 4 to 8 mice per experiment. Body and liver weights were measured upon dissection, and oil red O staining was performed in liver sections as previously described (Mehlem *et al*., 2013). Blood was collected during euthanasia, but samples were hemolyzed in the majority of animals. Animals were sacrificed using carbon dioxide after a 6 h fast.

### Hematoxylin and eosin staining

H&E completed by Baylor College of Medicine Comparative Pathology Core laboratory from formalin fixed and paraffin‐embedded tissue. The livers were fixed overnight in buffered 10% formaldehyde, processed and embedded in paraffin, and sectioned at a thickness of 4 μm. The sections were stained with H&E using a standard protocol.

### Oil red O staining

Oil red O staining was completed by Baylor College of Medicine Comparative Pathology Core laboratory. Briefly, frozen fresh liver tissue sections 7 μm thick were placed in propylene glycol and incubated for 6 min and then transferred to an 85% propylene glycol and distilled water mixture. The slides were then rinsed twice in distilled water and incubated in hematoxylin for 1‐2 min. The slides were washed with tap water then rinsed in distilled water before mounting and cover‐slipping.

### Liver triglycerides

Triglycerides in liver were measured at the Mouse metabolic Phenotyping Center (MMPC) at Vanderbilt University. Briefly, liver pieces (100 mg each) were homogenized in 0.5 mL of buffer (10 mm Tris, 150 mm NaCl, 1 mm EDTA, pH 7.5). Portions (300 μL) were transferred into a glass tube containing 600 μL of a 1:1 chloroform:methanol solution, mixed vigorously by vortex, and centrifuged at 960g for 10 min at 4C. After removal of the upper layer, the samples were dried with flowing N_2_ and resuspended in 100 μL of 5% triton X‐100 solution in extraction buffer. Ten μL aliquots were transferred into new tubes, and a standard curve was prepared with the use of different concentrations of triolein. Three hundred μL of triglyceride reagent (Sigma‐Aldrich, St. Louis, MO, USA) was added to the tubes containing samples and standards. After 30‐min incubation at room temperature, the samples were transferred to a microtiter plate and absorbance at 540 nm was measured. Triglyceride concentrations were expressed by mg of tissue protein measured by the Bradford assay.

### Western blot

Mouse livers from young (100 days old) and old (600 days old) animals were homogenized in buffer A containing 20 mm Tris‐HCl, pH 7.5, 30 mm KCl, 10 mm ‐mercaptoethanol, and inhibitors of phosphatases. Nuclei were spun down at 15 300g for 15 min, and supernatant (cytoplasm) was kept in the 80 °C freezer. The pellet (nuclei) was treated with buffer B containing 20 mm Tris‐HCl, pH 7.5, 0.42 m NaCl, 10 mm mercaptoethanol, 25% sucrose, 5 mm MgCl2, and inhibitors of phosphatases. After 30‐min incubation on ice, nuclei were spun down at 12 000 rpm for 10 min, and supernatant (nuclear extract) was frozen and kept in a 80 °C. 50 g of proteins was loaded on gradient 8–16% or 4–20% SDS–polyacrylamide gel (Bio‐Rad, Hercules, California 94547 USA). Proteins were transferred on nitrocellulose membrane, and the membrane was blocked with 10% dry milk on TTBS for 1 h. The membrane was incubated with primary antibodies for 2–4 h, washed, and incubated with secondary antibodies for 1 h. After wash, the signals were detected by detection reagents (Amersham Biosciences, Pittsburgh, PA 15264‐3065, USA). Protein loading was verified by a reprobe of the membranes with ‐CRT and by Coomassie stain. The following antibodies were used in Western blots: C/EBPα (14AA) (Santa Cruz Biotechnology, Dallas, TX, USA), calreticulin (1H2) (Santa Cruz Biotech), DGAT1 (H‐70) (Santa Cruz Biotechnology), GPAT1 (ProSci, Inc, Poway,CA, USA), P300 (N‐15 or C‐20) (Santa Cruz Biotechnology,), ATGL [EPR3444(2)] (Abcam, Eugene, OR, USA), Calreticulin (ProSci, Inc), PPARα (126285) (Abcam), FAS (F9554), (Sigma, St. Louis, MO, USA), LPL (F‐1), (Santa Cruz Biotechnology, Dallas, TX), β Actin (ProSci, Inc), and SCD‐1 [CD.E10], (Abcam). These antibodies as those used for other procedures have been previously validated by our group and others (Jin *et al*., 2013, 2016).

### Chromatin Immunoprecipitation assay (ChIP)

ChIP assays were performed using ChIP It kit as described previously (*Jin et al*., 2009; *Wang et al.,* 2007; *Wang et al*., 2008b). Briefly, chromatin solutions were prepared from livers of young and old ghrelin wild‐type and ghrelin knockout mice. C/EBPα and p300 were immunoprecipitated from the solution. DNA was isolated and used for the PCRs with primers covering C/EBPα sites. PCR mixtures were amplified for one cycle of 95 °C (5 min), 58 °C (5 min), and 72 °C (2 min), and then PCR mixtures were amplified for 31 cycles of 95 °C (1 min), 58 °C (2 min), and 72 °C (1.5 min). PCR products were separated by 2% agarose gel electrophoresis or by 8% polyacrylamide gel electrophoresis.

### Size exclusion chromatography (SEC)

This procedure was used for the isolation of C/EBPα. Nuclear extracts were isolated from livers as described earlier (Timchenko *et al*., 1997) and fractionated by size‐exclusion column SEC400 (HPLC, HR; Bio‐Rad Laboratories Inc, Hercules, CA USA). The detailed procedure for the analysis of C/EBPα complexes is described in our previous papers (Wang *et al*., 2001, 2004). Briefly, gel filtration fractions were loaded on denaturing gradient (4‐20%) PAAG, blotted onto membrane, and probed with antibodies to C/EBPα (14AA), HDAC1, HP1α, and Brm (Santa Cruz Biotechnology, Dallas, Texas+9), to detect C/EBPα complexes, C/EBPα was immunoprecipitated from each fraction, and IPs were probed with antibodies to HDAC1.

### Statistical analysis

SPSS 18.00 software for Windows (SPSS Inc. Chicago, IL, USA) was used for all statistical analysis and graph. Parameters are expressed as Mean ± SEM. Statistical comparisons for categorical data were performed using ANOVA followed by Games‐Howell post hoc analyses. *P* values of 0.05 or smaller were considered statistically significant. For more details on methods, please see Appendix S1.

## Funding

This work was funded by The U.S. Dept of Veterans Affairs (MERIT grants BX002807 and CX000174) and NIH Grant AG040583 to JMG. This work was also supported NIH grant R01DK102597 (NT). Dr Guillory was supported by a training grant from the NIA (T32AG000183). We thank the University of Washington DERC (P30 DK017047) and NORC (P30 DK035816), Vanderbilt MMPC (supported in part by U24 DK59637) for their help. We thank Dr. Roy G. Smith for providing the ghrelin WT and KO animals.

## Author contributions

JMG, NT, and BG designed experiments and wrote the manuscript. BG, BA, NJ, PZ, and PI conducted experiments. JMG, NT, and BG analyzed data.

## Conflicts of interests

All authors declare no competing financial interests.

## Supporting information


**Fig. S1** Transcript levels of key mediators in fat metabolism.
**Fig. S2** Protein levels of key mediators in fat metabolism.

**Table S1** RT‐PCR primers.
**Appendix S1** Reverse transcription quantitative real‐time PCR of mRNA.
