# Supplementary material for: Ghrelin deletion protects against age‐associated hepatic steatosis by downregulating the C/EBPα‐p300/DGAT1 pathway
Source: Aging Cell. 2017 Oct 12;17(1):e12688. doi: 10.1111/acel.12688 (PMC5771394; doi:10.1111/acel.12688)
Supplement: Supplementary file 2 — Table S1 RT‐PCR primers. Appendix S1 Reverse transcription quantitative real‐time PCR of mRNA. [file ACEL-17-na-s002.docx]

**Appendix S1**

**Reverse transcription quantitative real-time PCR of mRNA** Total RNA was isolated from mouse liver tissues (30–60 mg), using the guanidinium method (Trizol, Invitrogen, Carlsbad, CA). Tissues were homogenized using the Bullet Blender (Next Advance, Averill Park, NY), 500 ng of total RNA was assessed using a Nanodrop ND-1000 spectrophotometer (Saveen and Werner AB, Sweden). Extracted RNA was reverse transcribed (QuantiTect Reverse Transcription Kit, Qiagen) to cDNA. Transcript levels were measured by real-time PCR (9700HT Sequence Detection System; Applied Biosystems). Primers and probes for real-time PCR amplification were selected using Primer Express Software (Applied Biosystems, **Supplemental Table 1).** The probe for target genes was labeled at the 5_ end with a reported dye 6-fluorescein amidite (**FAM 6**) and at the 3_ end with a quencher dye tetramethylrhodamine (**TAMRA-6**). The reporter and quencher dyes are in close proximity on the probe, resulting in suppression of reporter fluorescence. The probe–exon is designed to hybridize to a specific sequence within the PCR product. The 5_ - to 3_ cleave activity of the *Taq*DNApolymerase allows for separation of the reporter from close proximity of the quencher dye, resulting in fluorescence of the reporter dye. The resulting signal is measured at each amplification cycle on the ABI Sequence Detection System (Applied Biosystems), thus allowing the measurement of sample abundance in the linear phase of amplification. Target genes were amplified using aliquots of the same cDNA sample, and final quantification of each sample was achieved by coamplified relative standard curve.

**Supplemental Table 1. RT-PCR primers**

| gene | FWD | REV |
| --- | --- | --- |
| PPAR-α | 5’-CCAGTATTTAGGAAGCTGTCCTG-3’ | 5’CGTTGTGTGACATCCCGACAG-3’ |
| FAS | 5’-AGGATGTCAACAAGCCCAAG-3’ | 5’-ACAGAGGACAAGGCCACAAA-3’ |
| SCD1 | 5’-TGAAAGCTGAGAAGCTGGTG-3’ | 5’-CAGTGTGGCAGGATGAAG-3’ |
| LPL | 5’-TCTCCTGATGATGCGGATTT-3’ | 5’-CAACATGCCCATCTGGTTTC-3’ |
| ATGL | 5’-GAAGATCTCCATGTTCCCGAGGGAG-3’ | 5’-CCGCTCGAGTCAGCAAGGCGGGAG-3’ |
| GAPDH | 5′-ACCACCATGGAGAAGGCCGG-3′ | 5′-CTCAGTGTAGCCCAAGATGC-3′ |
